# Supplementary material for: A liquid metal-based module emulating the intelligent preying logic of flytrap
Source: Nat Commun. 2024 Apr 22;15:3398. doi: 10.1038/s41467-024-47791-7 (PMC11035631; doi:10.1038/s41467-024-47791-7)
Supplement: Supplementary file 2 — Supplementary information [file 41467_2024_47791_MOESM2_ESM.pdf]

Supplementary Information for

A Liquid Metal-based Module Emulating the Intelligent Preying Logic of Flytrap

Yuanyuan Yang,[1] Yajing Shen[2,3,*]

[1]School of Aerospace Engineering, Xiamen University, Xiamen, China
[2]Department of Electronic and Computer Engineering, The Hong Kong University of Science and Technology, Clear Water Bay, Hong Kong, China
[3]Center for Smart Manufacturing, The Hong Kong University of Science and Technology, Clear Water Bay, Hong Kong, China

*Yajing Shen
Email: eeyajing@ust.hk

This PDF file includes:

Supplementary Figure S1: force analysis diagrams of extending liquid metal.

Supplementary Figure S2: the extending speed of liquid metal filament under different gate voltages.

Supplementary Figure S3: the extending speed of liquid metal filament with different volume.

Supplementary Figure S4: the extending speed of liquid metal filament within different channels.

Supplementary Figure S5: the digital image of the extending and connecting process.

Supplementary Figure S6: the working performance of device with different reservoirs.

Supplementary Figure S7: the conducted current with different molarity of NaOH.

Supplementary Figure S8: the remaining state of liquid metal filament after gate voltage removed.

Supplementary Figure S9: the structure and working mechanism of artificial sensory hair.

Supplementary Figure S10: fabrication process of electric actuator.

Supplementary Figure S11: bending mechanism of artificial petal.

Supplementary Figure S12: bending performance of electric actuator under different voltage applied.

Supplementary Figure S13: the response of LLM and artificial petal when long-time trigger applied.

Supplementary Figure S14: the schematic of the potential neuromorphic applications of LLM.

Supplementary Note: working mechanism of artificial flytrap.

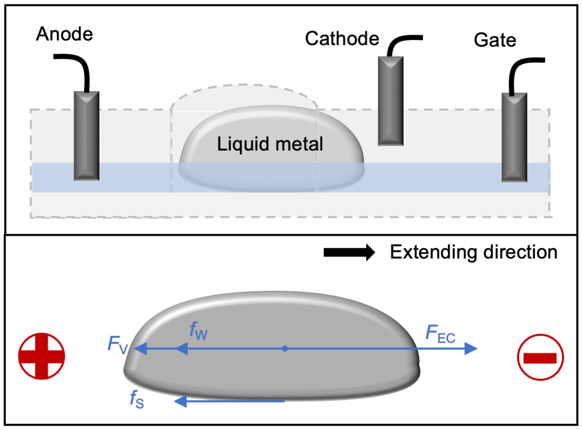

Supplementary Figure 1. Force analysis diagrams of extending liquid metal. F_{EC} is the driving force from the electrochemical effect-induced surface tension variation, F_V is the viscous force from surrounding electrolyte, f_S is the friction between the liquid metal and the substrate, and f_W is the friction between the liquid metal and the channel wall.

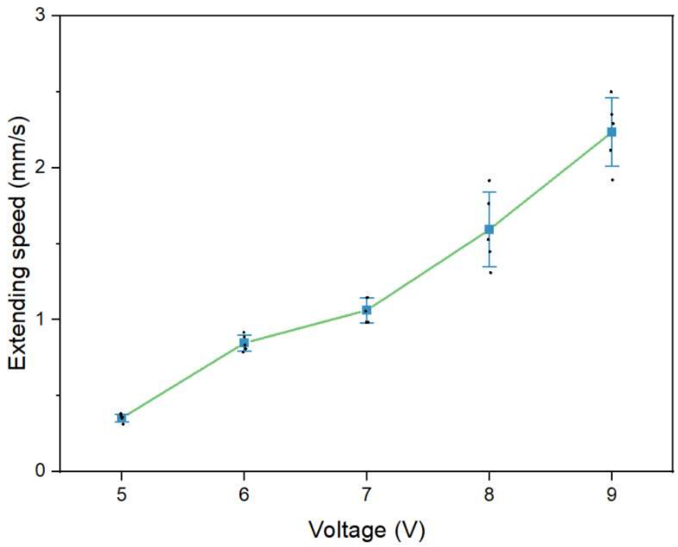

Supplementary Figure 2. The extending speed of liquid metal filament under different gate voltage triggers within 5 mm extending distance. Since the electrochemical effect-induced surface tension reduction is positively correlated to the voltage, the extending pressure P_{LM} as well as the extending speed of the liquid metal v_l are positively related to the voltage. Data are presented as mean values ± standard deviation (SD), the number of independent experiments n = 5.

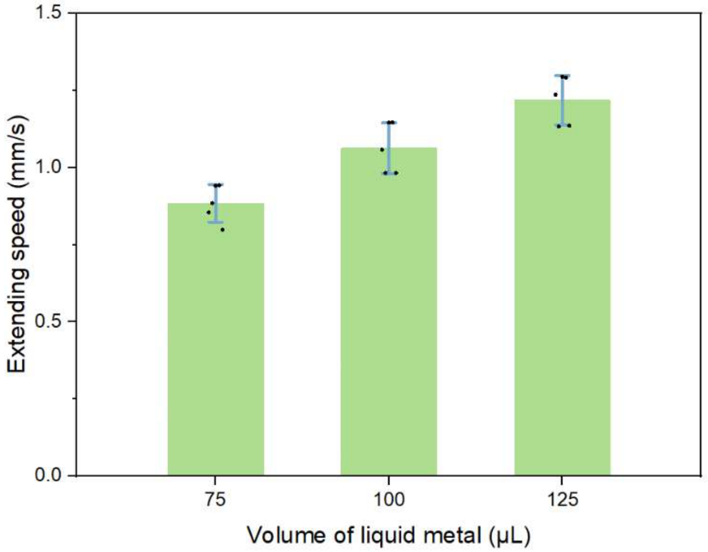

Supplementary Figure 3. The extending speed of liquid metal filament with different liquid metal volume. Since larger amount of liquid metal processes larger oxidation area, the surface tension difference would be larger, leading to higher pressure. Thus, the liquid metal with larger volume owns higher extending speed. Data are presented as mean values ± SD, the number of independent experiments n = 5.

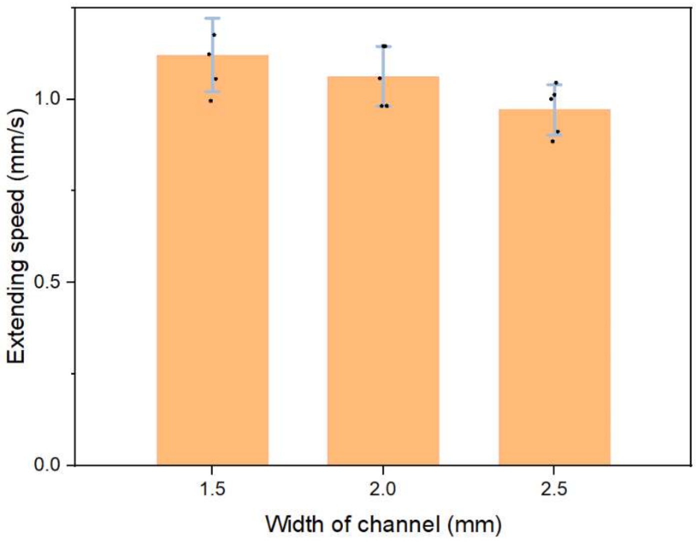

Supplementary Figure 4. The extending speed of liquid metal filament within different channels. Before extending to different channels, the liquid metal stays in the reservoir, of which the pressure and potential flow could be assumed as the same. Suppose droplets of the same mass flow through different channels with cross-sectional areas S_1 and S_2 at velocities v_1 and v_2 respectively, S_1v_1 should be equal to S_2v_2. When $S_1 < S_2$, $v_1 > v_2$. Therefore, the extending speed of liquid metal filament is smaller when liquid metal is placed in wider channel. Data are presented as mean values ± SD, the number of independent experiments n = 5.

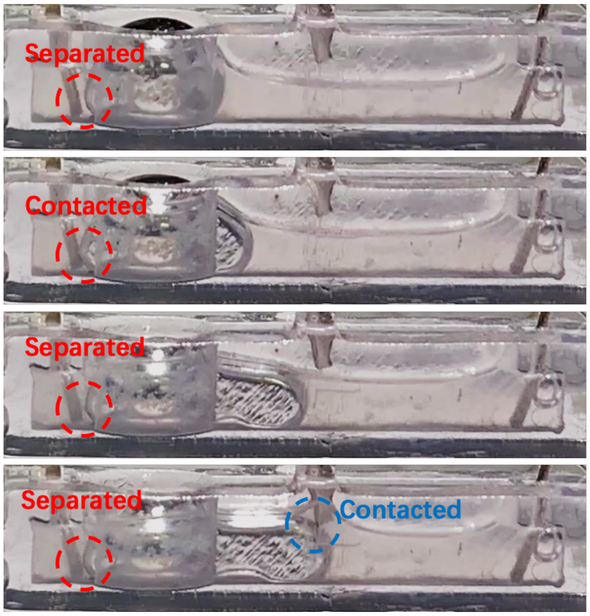

Supplementary Figure 5. The digital image of the extending and connecting process of liquid metal filament. The liquid metal is in contact with the anode at the first few seconds when voltage is applied on gate and anode. Since the volume of liquid metal is constant, during the extending process of liquid metal filament, the left end of liquid metal would gradually separate with the anode. When the cathode is conducted, the liquid metal is not directly in contact with the anode while directly contacts the cathode.

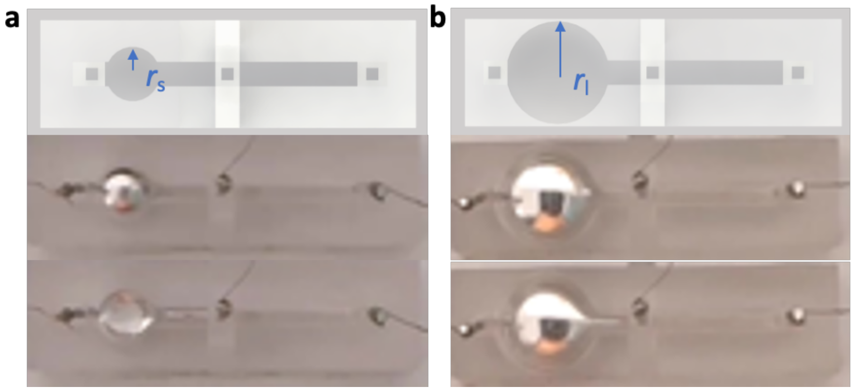

Supplementary Figure 6. The actuation performance of device with dimensions of reservoir, including a) radius r_s = 1.2 mm, and b) r_l = 3.2 mm. The experiment results show that the liquid metal could be manipulated controllably as well.

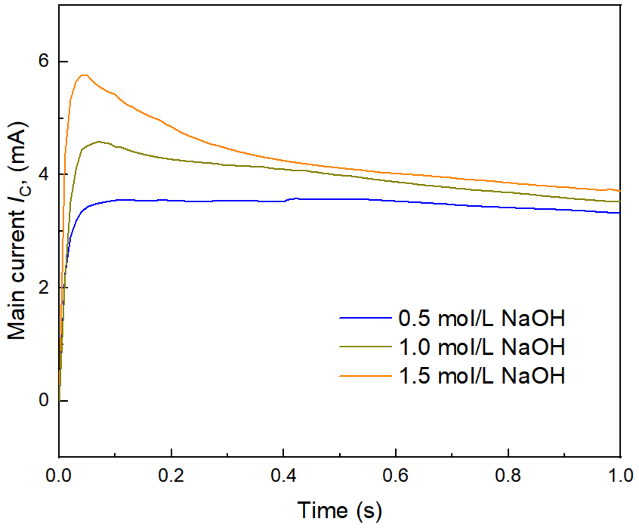

Supplementary Figure 7. The conducted current with different molarity of NaOH. Since the liquid metal is not direct connected to the anode, the conducted current would flow through NaOH as well. Thus, the current is positively related to the molarity of NaOH.

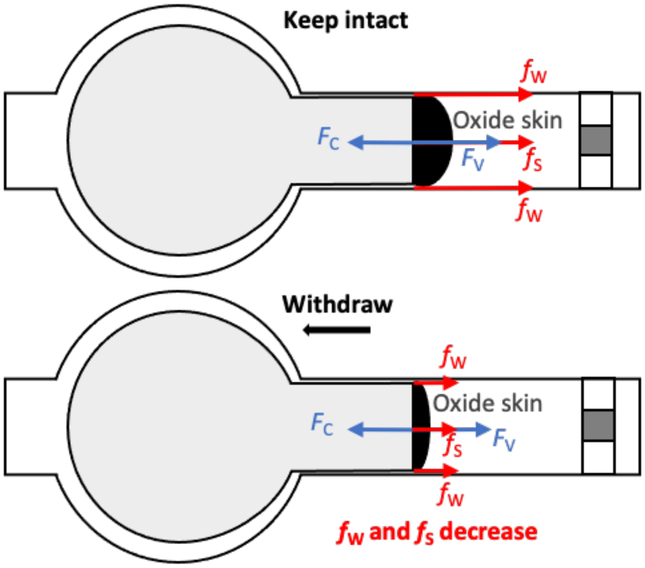

Supplementary Figure 8. The adhesion of oxide skin to channel-induced remaining state of liquid metal filament after the gate voltage is removed. F_C is the cohesive force of liquid metal, F_V is the viscous force from surrounding electrolyte, f_S is the friction between the liquid metal and the substrate, and f_W is the friction between the liquid metal and the channel wall. When V_G is turned off, the adhesion of the oxide film to channel will lead to a large friction force f_W and f_S that the liquid metal filament keeps intact. As the adhesion decreases over time, F_C would overcome the resistance leading to the withdraw of liquid metal filament.

Supplementary Note

Working mechanism of artificial flytrap

The artificial flytrap contains an electric switch-based artificial sensory hair and a soft electric actuator-based artificial petal. The electric switch is composed of Cu substrate, PDMS hair, and a Cu sheet as shown Supplementary Fig. 9. When the PDMS receives the mechanical trigger, it would bend to make the Cu sheet contact with the Cu substrate. In this case, the electric switch is ON with current flow through. The artificial petal is composed of silver nanowires (Ag NWs) - platinum (Pt) - paper/ polydimethylsiloxane (PDMS) bilayer soft actuator, which could bend/unbend according to the applied DC voltage. The fabrication process is shown in Supplementary Fig. 10. The bending mechanism is mainly based on the thermal expansion of PDMS layer as depicted in Supplementary Fig. 11. To be specific, when applying the DC voltage on the Ag NWs, the generated electrical current is converted into thermal energy via Joule heating, rapidly heating the conductive film. The produced thermal energy is then transferred to paper and PDMS layer. Since PDMS owns high coefficient of thermal expansion (CTE) while paper owns a very low CTE value, the bilayer would bend due to the interfacial stress. Once the applied voltage is turned off, the actuator gradually returns to its original shape due to the heat loss. The bending angle of actuator is related to the voltage applied that higher voltage would generate higher heat and thermal expansion volume, leading to the higher bending angle as shown in Supplementary Fig. 12.

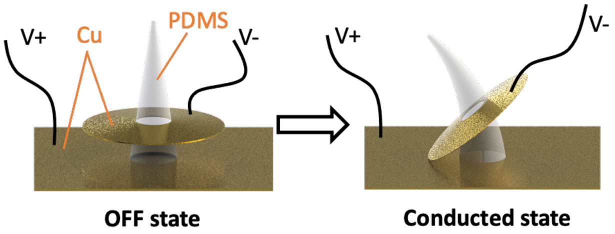

Supplementary Figure 9. Structure and working mechanism of electric switch-based artificial sensory hair. The electric switch is composed of Cu substrate, PDMS hair, and a Cu sheet. When the PDMS receives the mechanical trigger, it would bend to make the Cu sheet contact with the Cu substrate. In this case, the electric switch is ON with current flow through.

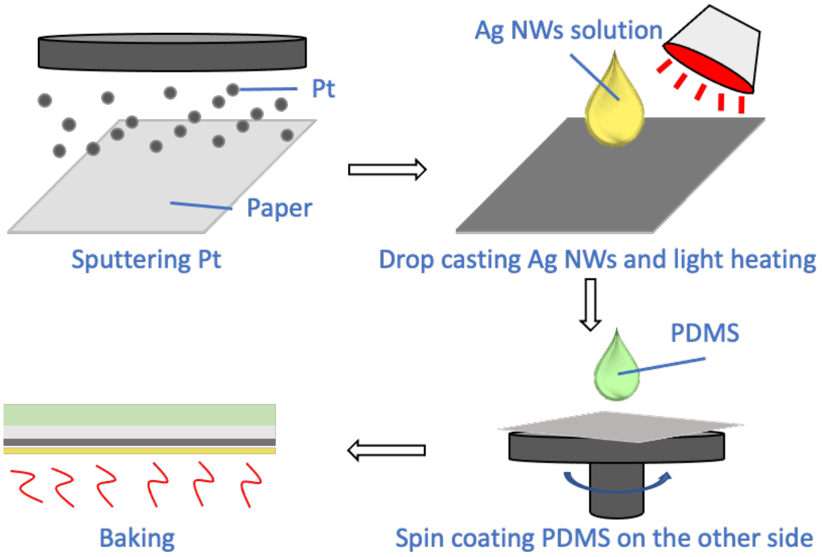

Supplementary Figure 10. Fabrication process of electric actuator. First, the paper sheet was coated with Pt layer using the sputtering system. The Ag NW solution was drop-cast on the Pt-coated paper sheet and dried under light heating. Then the PDMS was spinning coated on the opposite side of the obtained paper at the speed of 500 rpm for 15 s and 900 rpm for 30 s. After that, PDMS was cured at the temperature of 75 ºC for 2 h to obtain the Ag NWs-Pt-paper/PDMS composite film. The artificial flower petal can then be fabricated by cutting with a specific shape.

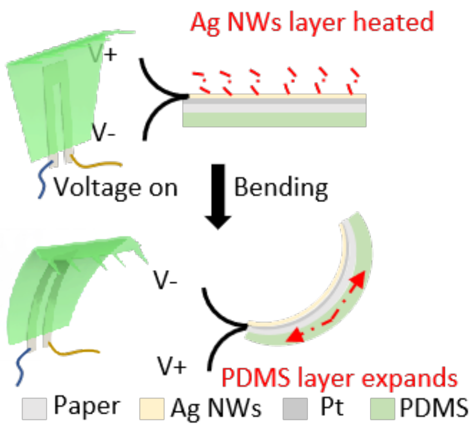

Supplementary Figure 11. Bending mechanism of artificial petal. When applying the DC voltage on the Ag NWs, the generated electrical current is converted into thermal energy via Joule heating, rapidly heating the conductive film. The produced thermal energy is then transferred to paper and PDMS layer. Since PDMS owns high CTE while paper owns a very low CTE value, the bilayer would bend due to the interfacial stress. Once the applied voltage is turned off, the actuator gradually returns to its original shape due to the heat loss.

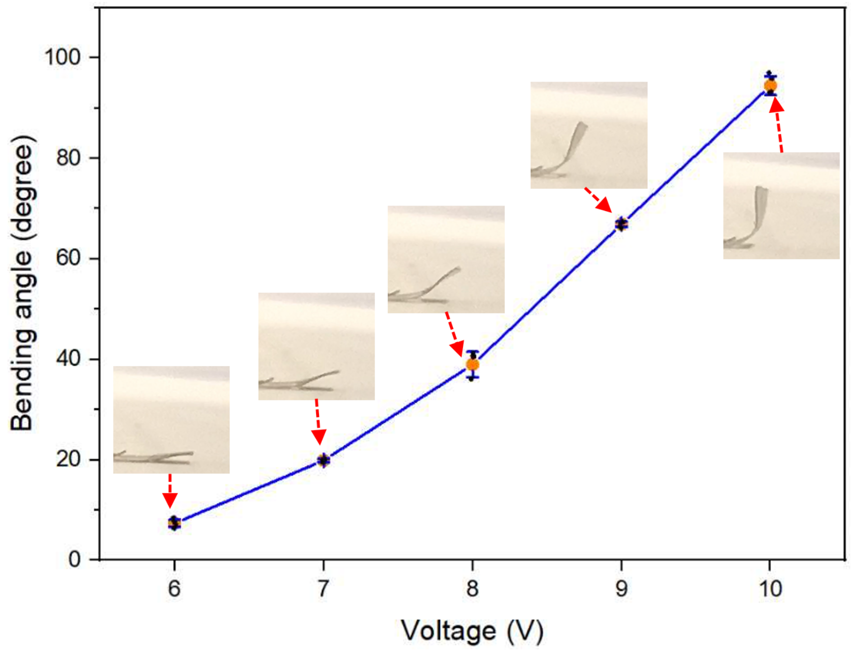

Supplementary Figure 12. The bending performance of electric actuator according to voltage applied. Higher voltage would generate higher heat and thermal expansion volume, leading to the higher bending angle (sample size: 5*15 mm). Data are presented as mean values ± SD, the number of independent experiments n = 5.

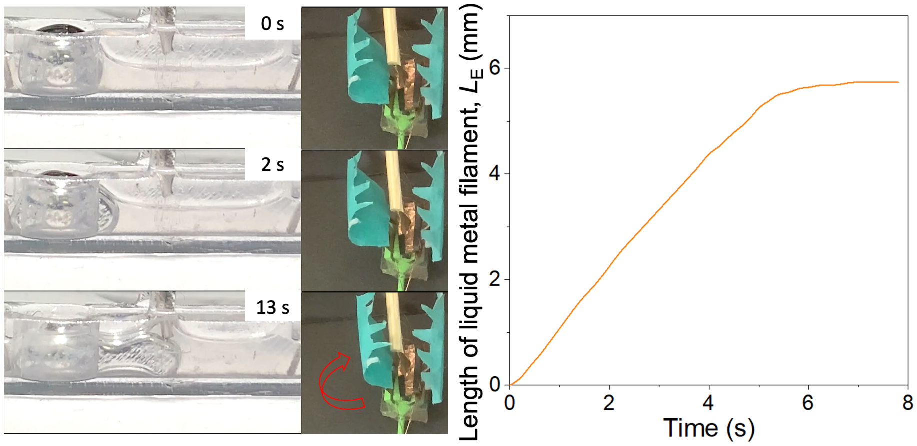

Supplementary Figure 13. The response of LLM and artificial petal when long-time trigger is applied. When a mechanical trigger of long duration (t = 6 s) is applied to the electric switch, the V_G of the LLM undergoes a change to 7 V, resulting in the extension and connection of the liquid metal to the cathode. Consequently, the output signal manifests as current flowing from the anode to the cathode, thereby actuating the artificial petal to bend and close

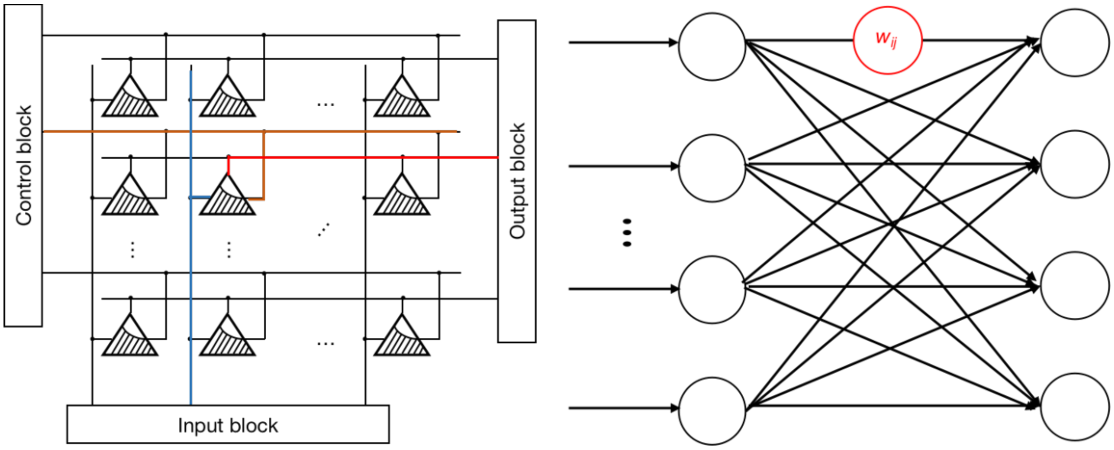

Supplementary Figure 14. The schematic of the potential neuromorphic applications of LLM. The LLMs could be placed at the junctions of crossbar to carry out logic operations. The outputs of the crossbar depend on the applied voltages to the rows and the columns of the crossbar. Due to the integrated memristor and transistor function of LLM, storage and computation can both be carried out. The crossbar nodes' conducting state could then be used as the weights of neural network for training.
