# Supplementary material for: A liquid metal-based module emulating the intelligent preying logic of flytrap
Source: Nat Commun. 2024 Apr 22;15:3398. doi: 10.1038/s41467-024-47791-7 (PMC11035631; doi:10.1038/s41467-024-47791-7)
Supplement: Supplementary file 3 — Description of additional supplementary files [file 41467_2024_47791_MOESM3_ESM.pdf]

Description of Additional Supplementary Files

File name: Supplementary Movie 1

Description: The response of flytrap to different triggers. The movie shows that the Venus flytrap uses two intelligent strategies to distinguish the prey from other disturbances and generate enough electrical signals to close its trap, i.e., fast consecutive double touches and long single touch.

File name: Supplementary Movie 2

Description: The structure and dynamic response of liquid metal-based logic module (LLM) to different triggers. The movie shows that LLM mainly consists of a mushroom-shaped channel with a reservoir and a connected pathway. Liquid metal is placed in the reservoir and the rest of the cavity is filled with NaOH solution. Three Pt electrodes are inserted as the anode, cathode, and gate respectively. When the two short-time triggers occur within a time period $\Delta t < \Delta tr$, the liquid metal filament elongates and connects the cathode. On the other hand, when $\Delta t > \Delta tr$, the liquid metal filament would fail to connect the cathode.

File name: Supplementary Movie 3

Description: The structure of artificial flytrap and its response to different triggers. The movie shows that the artificial flytrap comprises a voltage supply, an electric switch-based artificial sensory hair, and a soft electric actuator-based artificial petal, all of which are connected to the anode, gate, and cathode of the liquid metal-based logic module (LLM), respectively. Both the single long-time trigger and double short-time triggers within threshold time could lead to the bend of artificial petal.

File name: Supplementary Movie 4

Description: The potential applications of liquid metal-based logic module (LLM). The movie shows that the LLM could serve as a functional equivalent to an integrated memristor/transistor, which holds potentials for a wide range of applications, i.e., integrated high-pass filter by utilizing moving window statistics and synapse of neural networks.
